# KIR2DS5 in the presence of HLA-C C2 protects against endometriosis

**DOI:** 10.1007/s00251-015-0828-3

**Published:** 2015-03-01

**Authors:** Izabela Nowak, Rafał Płoski, Ewa Barcz, Piotr Dziunycz, Paweł Kamiński, Grażyna Kostrzewa, Łukasz Milewski, Piotr I. Roszkowski, David Senitzer, Jacek Malejczyk, Piotr Kuśnierczyk

**Affiliations:** 1 Laboratory of Immunogenetics and Tissue Immunology, Department of Clinical Immunology, Ludwik Hirszfeld Institute of Immunology and Experimental Therapy, Polish Academy of Sciences, ul. Rudolfa Weigla 12, 53-114 Wrocław, Poland; 2 Department of Medical Genetics, Centre of Biostructure Research, Medical University of Warsaw, Kampus Banacha, ul. Pawińskiego 3c, 02-106 Warszawa, Poland; 3 First Chair and Clinic of Obstetrics and Gynecology, Medical University of Warsaw, Pl. Starynkiewcza 1/3, 02-015 Warszawa, Poland; 4 Department of Histology and Embryology, Centre of Biostructure Research, Medical University of Warsaw, ul. Chałubińskiego 5, 02-004 Warsaw, Poland; 5 Second Clinic of Obstetrics and Gynecology, Medical University of Warsaw, ul. Karowa 2, 00-315 Warszawa, Poland; 6 Laboratory of Histocompatibility, City of Hope, National Medical Center, 1500 East Duarte Road, Duarte, CA 91010 USA

**Keywords:** Endometriosis, Killer cell immunoglobulin-like receptor, Human leukocyte antigen, Linkage disequilibrium

## Abstract

Endometriosis is defined as the presence of functional endometrial tissue outside the uterine cavity. Several hypotheses have attempted to explain the etiology and pathogenesis of endometriosis. Recently, it has been suggested that a defect of the natural killer (NK) activity in the recognition and lysis of endometrial cells is one of the crucial points in the development of this disease. Natural killer cells can express killer immunoglobulin-like receptors (KIR), which recognize class I human leukocyte antigens on target cells. We asked whether polymorphisms in *KIR*, *HLA-C*, and *HLA-B* genes are risk factors for endometriosis. We tested 153 women with endometriosis diagnosed on the basis of laparoscopic and histological examination, and 213 control healthy women, who gave birth to at least one child. The frequency of *KIR* genes in patients was similar to that in controls except for *KIR2DS5*, which exerted a protective effect only in *HLA-C* C2-positive individuals. Moreover, *KIR2DS5-*positive women with endometriosis had 13 times lower chance that the disease would occupy the peritoneum than *KIR2DS5-* and *KIR2DS4del*-negative ones (OR = 0.077, *P* = 0.0061). Similarly, *KIR2DS4del*-positive endometriotic persons had 11 times lower chance for peritoneal disease (OR = 0.094, *P* < 0.001). Negative linkage disequilibrium between *KIR2DS5* and *KIR2DS4del* indicates that these genes are mutually exclusive. Our data suggest that *KIR2DS5* may be associated with protection from endometriosis, whereas *KIR2DS4del* seems to be associated with higher disease stages, possibly by exclusion of protective *KIR2DS5*.

**Electronic supplementary material:**

The online version of this article (doi:10.1007/s00251-015-0828-3) contains supplementary material, which is available to authorized users.

## Introduction

Endometriosis is a common gynecological disease, which affects approximately from 2 to 10 % of women in reproductive age. Patients with endometriosis suffer from pelvic pain, dyspareunia, dysuria, dysmenorrhea, and they constitute about 50 % of infertile women (Dunselman et al. 2014). Endometriosis is defined as the presence of functional endometrial tissue outside the uterine cavity, mostly in the pelvic peritoneum, ovaries, and rectovaginal septum (Burney and Giudice 2012; Gupta et al. 2008; The Practice Committee of the American Society for Reproductive Medicine 2012). Several hypotheses have attempted to explain the etiology and pathogenesis of endometriosis, mainly Sampson’s theory of retrograde menstruation of the endometrial tissue through the fallopian tubes and into the peritoneal cavity, and its subsequent implantation (Sampson 1927). It has been suggested that a defect of the natural killer (NK) activity in the recognition and lysis of these implanted endometrial cells may be one of the crucial points in the initiation and progression of endometriosis (Berkkanoglu and Arici 2003).

Natural killer cells can express killer immunoglobulin-like receptors (KIR), which recognize class I human leukocyte antigens (HLA) on target cells and may either stimulate or inhibit NK cell activity. KIR receptors occur as two or three extracellular immunoglobulin-like domain molecules with a long (L) or short (S) cytoplasmic tail. KIR2DL/KIR3DL has inhibitory function in contrast to KIR2DS/3DS with activating function. *KIR* genes exhibit extensive haplotypic and allelic polymorphism. Individuals differ in both the number and kind (activating vs. inhibitory) of *KIR* genes (Middleton and Gonzelez 2010; Falco et al. 2013; Kulkarni et al. 2008).

HLA-C molecules can be divided into two groups, HLA-C C1 and C2, according to the amino acid present at position 80 of the molecule. All HLA-C molecules with asparagine at position 80 belong to the HLA-C C1 group, and are recognized by KIR2DL2/2DS2 and KIR2DL3, whereas those with lysine in this position belong to the HLA-C C2 group and constitute ligands for KIR2DL1/2DS1. However, KIR2DL2/2DL3 may also bind to some C2 molecules. KIR2DS4 binds HLA-A11 and some C1 and C2 molecules (Graef et al. 2009). Finally, KIR3DL1 has specificity for HLA-Bw4 epitope (Middleton and Gonzelez 2010; Falco et al. 2013; Kulkarni et al. 2008).

*KIR* and *HLA* polymorphism affects NK cell reactivity and susceptibility to various diseases (Kulkarni et al. 2008). The aim of our study was to find an association of *KIR* genes and their *HLA* ligands with susceptibility to endometriosis in Polish women. To our knowledge, this is the first study in females emphasizing the role of *KIR2DS5* and *KIR2DS4* deletion variant (*KIR2DS4del*) in clinical stages of endometriosis.

## Materials and methods

### Study design

One hundred and fifty-three Polish patients with endometriosis (age range, 20–58 years; mean ± S.D., 33.7 ± 8.0 years) diagnosed at the First and Second Department of Obstetrics and Gynecology, Medical University of Warsaw for pelvic pain, dysmenorrhea, and/or infertility were included in the study. Patients were classified according to the revised American Fertility Society criteria (American Fertility Society 1985) and both laparoscopic and histopathological examinations. All patients were categorized into four subgroups: I–II—minimal and mild stages, III—moderate, and IV—severe stages. Among them, 98 (64.1 %) patients manifested ovarian endometriosis, 43 (28.1 %) patients had ovarian and peritoneal endometriosis, and 12 (7.8 %) patients had only peritoneal endometriosis.

The control group consisted of 213 women. Among this group, 96 women were anonymous adults from paternity testing, who gave birth to at least one child, but there was no information on the disease status as they were anonymous. The remaining 117 women (age range, 23–68 years; mean age ± S.D., 31.37 ± 6.36) reported having at least two babies with the same partner and no gynecological, endocrinological, or immunological disorders (described earlier by Nowak et al. 2009). Experimental protocols were approved by the Local Ethics Committee and all patients gave their informed consent to the study.

### DNA preparation and genotyping

Genomic DNA was isolated from venous blood using the salting out method or using an Invisorb Spin Blood Midi Kit (Invitek, Berlin, Germany) following the manufacturer’s instructions. *KIR* genotyping was described previously by Nowak et al. (2009) and Sun et al. (2004). Our *KIR* typing is validated three times a year by the International KIR Exchange Program organized by the Immunogenetics Center of the University of California at Los Angeles.

*HLA-C* gene fragments determining the *HLA-C1* and *C2* groups were distinguished according to a PCR-SSP method described by Frohn et al. (1998). *HLA-Bw4* and *HLA-Bw6* alleles were genotyped as described in Supplement 1.

### Statistical analysis

The proportion of individuals positive for a given *KIR* gene as well as *HLA-C1* and *C2* groups and *HLA-Bw4* was established by direct counting. *KIR* gene frequencies (gf) were estimated using the formula gf = 1 − √(1 − pf), where pf is the proportion of the population positive for the gene. Odds ratio and its 95 % confidence interval were used as a measure of effect size. Generalized linear models with binomial errors and the chi-square test for trend were used to investigate the relationship between clinical and genetic variables. Akaike’s information criterion was used as a measure of fit of models. Confidence intervals were estimated with the *bootstrap* approach. *P* values were computed exactly by numerical simulations. Measures for the estimation of linkage disequilibrium (LD) were the correlation of two alleles’ frequencies, *r*, global squared correlation between two loci, *R* ^2^ and Kullback-Leibler divergence of two loci from LE (Excoffier and Slatkin 1995; Abdallah et al. 2003). For the two loci *2DS4* and *2DS5*, *r* and *R* ^2^ were obtained as: $r=\frac{D_{ij}}{\sqrt{p_i{q}_j}}$, where *p*_*i*_ and *q*_*j*_ are the population allele frequencies of the *i*th allele on locus 2DS4 and the *j*th allele on locus *2DS5, D*_*ij*_ = *x*_*ij*_ − *p*_*i*_ *q*_*j*_, and *x*_*ij*_ is the frequency of the haplotype with alleles *i* and *j* on loci *2DS4* and *2DS5*, respectively. ${R}^2={\displaystyle {\sum}_i^3{\displaystyle {\sum}_j^2\frac{D_{ij}^2}{p_i{q}_j}.}}$ Kullback-Leibler divergence (Bhasi et al. 2006; Liu and Lin 2005), *D*_KL_ is a measure of distance between the observed haplotype distribution and the expected distribution assuming LE: ${D}_{\mathrm{KL}}={\displaystyle {\sum}_i^3{\displaystyle {\sum}_j^2{x}_{ij} log\frac{x_{ij}}{p_i{q}_j}.}}$ The chi-square statistic was calculated to test whether all of the *D*_*ij*_’s between 2DS4 and 2DS5 are zeros: ${\chi}_{df=2}^2={\displaystyle {\sum}_i^3{\displaystyle {\sum}_j^2\frac{n{D}_{ij}^2}{p_i{q}_j}.}}$

## Results

The frequencies of *KIR* genes in patients were similar to the frequencies observed in control women (Table 1) except for *KIR2DS5*. This latter result has already been described, but without analysis of other *KIR* genes (Nowak et al. 2010). The presence of *KIR2DS5* significantly decreased the risk of endometriosis. The *KIR2DS5* effect was independent from other *KIR* genes, including *KIR2DS4* (*P* = 0.181). However, it was seen only in *HLA-C C2*-positive individuals: in this group, those who possessed *KIR2DS5* had 2.5 lower chance of getting endometriosis than KIR2DS5-negative women (Supplement 2). In contrast, *KIR2DS5* presence did not affect the risk of disease in *C2*-negative individuals (Supplement 2). Noteworthy, *C2* presence or absence itself had no relation to disease risk (*P* = 0.363). Similarly, there was no correlation of *HLA-Bw4* or *HLA-Bw6* with disease (data not shown).

**Table 1:** *KIR* gene frequencies in patients and controls

Group	KIR gene												
	2DL1	2DL2	2DS3	2DL3	2DS2	3DL1	3DS1	2DL5	2DS1	2DS4fl	2DS4del	2DS4	2DS5
Patients, *N*	147	89	61	135	91	147	51	79	60	51	125	147	33*
gf^a^	80.3	35.3	22.5	65.6	36.4	80.3	18.3	30.4	22.0	18.3	57.2	80.3	11.5
Controls, *N*	205	115	70	188	116	201	85	113	94	61	181	201	68
gf	80.5	32.2	18.1	65.8	32.5	76.3	22.5	31.4	25.2	15.5	61.3	76.3	17.5
OR	0.96	1.19	1.35	1.0	1.23	1.46	0.75	0.94	0.82	1.25	0.79	1.46	0.58

*N* number of cases, *p* probability, *OR* odds ratio, *95 % CI* confidence interval

Patients vs controls, *P = 0.033, 95 % CI = 0.36–0.95

^a^gf, gene frequency calculated according to the formula gf = 1 − √(1 − pf)

We also analyzed the genetics of peritoneal versus ovarian endometriosis (Table 2). *KIR2DS5*-positive, *KIR2DS4del*-negative women with endometriosis had 13 times lower chance that the disease would occupy the peritoneum than *KIR2DS5-* and *KIR2DS4del*-negative ones (OR = 0.077, *P* = 0.0061). The percentage of individuals with peritoneal endometriosis in *KIR2DS5-*positive, *KIR2DS4del*-negative persons was only 25 % in comparison to 81 % in the double-negative group. Similarly, *KIR2DS5-*negative, *KIR2DS4del*-positive, endometriotic persons had lower (almost 11 times) chance for peritoneal disease in relation to double negative ones (OR = 0.094, *P* < 0.001). Interestingly, although double positive (*KIR2DS5* ^+^, *KIR2DS4del* ^+^) genotype had also a decreased chance of peritoneal endometriosis, in this case the protective effect was weaker (Table 2): this form of disease was present in nearly 43 % of patients with this genotype. *KIR2DS4-full-length* gene presence was not related to severity of disease (data not shown). *KIR2DS5* and *KIR2DS4* genes are in a strong negative linkage disequilibrium, as reflected by a complete lack of haplotype positive both for *KIR2DS5* and *KIR2DS4del* (more frequent variant of *KIR2DS4*) in the control group (Table 3). This haplotype is also absent from women with ovarian endometriosis, but present in 4 % of patients with peritoneal form of disease (Table 3).

**Table 2:** Chance of peritoneal endometriosis depending on *KIR2DS5/2DS4del* genotype

2DS5	2DS4del	Peritoneum		Number	Percent	OR	95 % CI		*P* value
		+	−						
−	−	13	3	16	81.25	1	–	–	–
+	+	9	12	21	42.86	0.173	0.038	0.795	0.041
+	−	3	9	12	25.00	0.077	0.012	0.478	0.0061
−	+	30	74	104	28.85	0.094	0.025	0.356	<0.001

Two-sided Fisher’s exact test was used to estimate differences between patients with peritoneal and non-peritoneal endometriosis

*N* number of cases with relevant genotype, *p* probability, *OR* odds ratio, *95 % CI* confidence interval

**Table 3:** Linkage disequilibrium between two loci: *KIR2DS4* and *KIR2DS5*

Group	Haplotypes	2DS4*full*/2DS5	2DS4*del*/2DS5	―/2DS5	2DS4*full*/―	2DS4*del*/―	―/―
Controls	HFs	0.0019	0	0.1724	0.153	0.6172	0.0555
	*r*	−0.153	−0.328	0.666	0.07	0.151	−0.306
	*R* ^2^ = 0.6952 *D*_KL_ = 0.4701			*χ* ^2^ = 148.77; df = 2; *P* < 0.00001			
Peritoneum+	HFs	0	0.0422	0.0867	0.1976	0.5245	0.149
	*r*	−0.16	−0.114	0.323	0.061	0.044	−0.124
	*R* ^2^ = 0.1639 *D*_KL_ = 0.1473			*χ* ^2^ = 9.178; df = 2; *P* = 0.01016			
Peritoneum−	HFs	0.0044	0	0.1077	0.15	0.6707	0.0671
	*r*	−0.098	−0.274	0.629	0.035	0.097	−0.224
	*R* ^2^ = 0.5413 *D*_KL_ = 0.3093			*χ* ^2^ = 53.59; df = 2; *P* < 0.00001			

*HFs* haplotype frequencies, *D*_*KL*_ Kullback-Leibler divergence from linkage equilibrium, *R* ^*2*^ global squared correlation coefficient for two loci, *r* correlation coefficient for allele frequencies, ― lack of a given gene (*KIR2DS4* and/or *KIR2DS5*), *Peritoneum+* and *Peritoneum−*, the presence or absence of peritoneal endometriosis

Last but not least, the severity of disease seemed to be associated with the *KIR2DS4del* gene, as the frequency of *KIR2DS4del* was increased in higher disease stages (Table 4). No other genes had any effect on the severity of endometriosis (data not shown).

**Table 4:** Frequency of *KIR2DS4del* positivity depending on endometriosis stage

Group	Disease stage		
	I + II	III	IV
2DS4del−	5	9	6
2DS4del+	7	33	49
%	58.33	78.57	89.09
*P*	0.012		

The chi-square test for trend was used to investigate the relationship between clinical and genetic variables

*p* probability, *I + II* minimal and mild endometriosis, *III* moderate, *IV* severe

## Discussion

Endometriosis is an enigmatic disease, in which many factors—genetic (Montgomery et al. 2008), neuroendocrine (Tariverdian et al. 2007), immunological (Tariverdian et al. 2007; Matarese et al. 2003)—are involved in the pathogenesis. The most accepted “transplantation theory” (Sampson 1927) explains the presence of the majority of endometriotic lesions in the peritoneal cavity by insufficient immune surveillance. Retrograde menstruation happens in up to 90 % of women in reproductive age. However, only minority of these women develop endometriosis. Therefore, it is generally accepted that changes in the activity of immune cells in the peritoneal microenvironment play an important role in the etiology of this disease (Berkkanoglu and Arici 2003; Matarese et al. 2003; Dmowski and Braun 2004; Ulukus et al. 2006). Particularly, it has been shown that NK cells of peripheral blood and peritoneal fluid of endometriotic patients exhibit decreased cytotoxicity to autologous endometrium (Oosterlynck et al. 1991; 1992). This impaired activity also correlated with severity of the disease. Moreover, Japanese scientists found an increased percentage of KIR2DL1-positive NK cells in peritoneal fluid and peripheral blood of women with pelvic endometriosis, which suggests stronger inhibition of NK cell activity against ectopic endometrium (Maeda et al. 2002). Individual NK cells differ in their KIR repertoire because this results from stochastic KIR expression in individual clones (Parham 2005). Nevertheless, a high degree of genotypic polymorphism (i.e., the presence or absence of particular *KIR* genes in the genotype of a given individual) allows NK cells to express only those *KIR* genes which are present in their genotype. Therefore, we looked for possible associations of *KIR* genes with susceptibility to endometriosis, localization of lesions (ovarian versus peritoneal), and severity of the disease.

We previously described decreased frequency of *KIR2DS5* in patients with endometriosis in comparison to healthy control (Nowak et al. 2010) but other *KIR* genes were not analyzed there. Here, we report the protective effect of *KIR2DS5* only in females who possessed the *HLA-C* C2 group. The *KIR2DS5* gene has been proven to be expressed at both the mRNA (Della Chiesa et al. 2008) and protein (Steiner et al. 2014) level, although it has been reported that its transcript could be detected in only 10 % of individuals positively genotyped for this gene (Leung et al. 2005), and protein expression of different alleles varies (Steiner et al. 2014). However, analysis of KIR expression at the protein level is difficult because of cross-reactivity of available antibodies, especially between inhibitory and activating receptors, which frequently have very similar amino acid sequences in their extracellular domains. Recently, it has been shown that monoclonal antibodies 143211 and HPMA4, which were previously thought to be specific exclusively for KIR2DL1 and KIR2DS1/L1, respectively, also bind KIR2DS5 (143211) or KIR2DS5 and KIR2DS3 (HPMA4) (Czaja et al. 2014). Therefore, studies showing increased expression of KIR2DL1 but not other KIRs in women with pelvic endometriosis (Maeda et al. 2002) should be treated with caution.

A physiologic ligand for KIR2DS5 is unknown (Parham et al. 2012). However, circumstantial evidence suggests that it may interact with both C1 and C2, because, in hematopoietic stem cell transplantation, leukemia-free survival and relapse rate depended on the presence or absence of the donor *KIR2DS5* gene as well as on the *C1/C2* versus *C1/C1* or *C2/C2* genotype of the recipient (van der Meer et al. 2008). Our finding, mentioned above, also suggests an interaction between KIR2DS5 and HLA-C molecules, influencing the ability of KIR2DS5+ NK cells to eliminate ectopic endometrium in C2+ women. Interestingly, *KIR2DS5* was found protective against another gynecological disorder, pre-eclampsia, in British Europeans and Ugandan Africans, and fetal *C2* inherited from the father increased the risk of disease in *KIR2DS5*-negative women (Nakimuli et al. 2015). However, only one allele, KIR2DS5*006, was protective in Ugandans, and this allele is absent from European populations, possessing only KIR2DS5*002 if any (Hou et al. 2009; Gonzalez-Galarza et al. 2015). Therefore, it might be that different *KIR2DS5* alleles affect pre-eclampsia and endometriosis.

Moreover, we observed preferential non-peritoneal localization of lesions in patients possessing either the *KIR2DS5* or *KIR2DS4del* gene, whereas simultaneous presence of both genes had a weaker effect. Interaction between these two genes has also been found recently in renal transplantation, where we detected a protective effect of the *KIR2DS5* gene against acute graft rejection, but only in the absence of *KIR2DS4del* (Nowak et al. 2012). Interestingly, the effect of the *KIR2DS4*-*full-length* gene on the graft protection associated with *KIR2DS5* was opposite, i.e., *KIR2DS5* was protective only if *KIR2DS4* full-length was present (Nowak et al. 2012). Here, we observed a much lower percentage of peritoneal endometriosis in women possessing either the *KIR2DS5* or *KIR2DS4del* gene than when both were present. Moreover, even in the subgroup of *KIR2DS5*+/*KIR2DS4del*+ patients, the fraction with peritoneal lesions was twice as low as in women negative for both these genes. However, the numbers were too small to draw a firm conclusion.

*KIR2DS5* and *KIR2DS4* genes are in strong negative linkage disequilibrium in our population (Table 3 and Niepiekło-Miniewska et al. 2013), and *KIR2DS4del* and *KIR2DS4-full-length* are alleles. Therefore, individuals positive for *KIR2DS5* and *KIR2DS4del* are highly unlikely to possess *KIR2DS4-full-length*; similarly, individuals positive for *KIR2DS5* and *KIR2DS4-full-length* are highly unlikely to be *KIR2DS4del*-positive. In peritoneal endometriosis, we observed only a weak effect of *KIR2DS4del* on *KIR2DS5* association with lack of endometrial lesions in the peritoneum, and no effect of *KIR2DS4-full-length. KIR2DS4-full-length* variant and *KIR2DS5* had frequencies of only 18.3 and 11.5 % in our patients, respectively, so the number of women with peritoneal endometriosis (*N* = 55) was perhaps too small to detect the interaction of *KIR2DS4-full-length* with *KIR2DS5* similar to that observed in kidney graft rejection.

Patients with endometriotic lesions limited to the peritoneum belong to a group of mild severity of this disease. NK cells may have the potential to remove endometriotic tissues from the peritoneal cavity but may not have such easy access to ovarian cysts, which are associated with more severe manifestation of endometriosis. Associations of *KIR2S5* and *KIR2DS4del* with protection from peritoneal but not ovarian lesions in endometriotic patients suggest that peritoneal endometriosis may have a different genetic background than ovarian endometriosis. Indeed, the same *KIR2DS4del* which was associated with protection from peritoneal lesions was associated with the highest grades of endometriosis which encompass ovarian lesions.

The only report on *KIR* gene associations with endometriosis published so far in addition to our *KIR2DS5* paper (Nowak et al. 2010) is that by Kitawaki et al. (2007), who described decreased frequency of *KIR3DS1* and increased frequency of highly inhibitory KIR-HLA combinations. This result is somewhat similar to ours, because *KIR* haplotypes consisting mostly of inhibitory *KIR* genes (called A haplotypes) are lacking *KIR2DS5* although not *KIR2DS4* (Jiang et al. 2012). In addition, we also observed slightly decreased presence of *KIR3DS1* in patients with endometriosis but it was not significant. The Japanese population demonstrates different *KIR* gene distribution from Europeans (allele frequency net: Gonzalez-Galarza et al. 2015), and is particularly rich in A haplotype homozygotes (Hiby et al. 2004).

In conclusion,

(i) The presence of *KIR2DS5* reduced the risk of disease if a female possessed alleles from group C2 of *HLA-C*.
(ii) In endometriotic patients, *KIR2DS5* or *KIR2DS4del* presence lowered the risk of localization of lesions in the peritoneum.
(iii) On the other hand, *KIR2DS4del* presence in patients was associated with severity of endometriosis, which, due to strong negative LD, may suggest an indirect effect of *KIR2DS5* absence.
(iv) Our results suggest a different genetic background of peritoneal and ovarian endometriosis.

Zhang et al. (2006) proposed KIR and HLA expression studies as an immunodiagnostic parameter for pelvic endometriosis. We believe that our results may support their proposal and extend it to genotyping patients for *KIR2DS4* and *KIR2DS5*, as well as for *HLA-C C1* and *C2*.

## Electronic supplementary material

Below is the link to the electronic supplementary material.

Supplement 1 (DOCX 14 kb)

Supplement 2 (DOCX 13 kb)
