# Supplementary material for: KIR2DS5 in the presence of HLA-C C2 protects against endometriosis
Source: Immunogenetics. 2015 Mar 1;67(4):203–9. doi: 10.1007/s00251-015-0828-3 (PMC4357646; doi:10.1007/s00251-015-0828-3)
Supplement: Supplementary file 1 — (DOCX 14 kb) [file 251_2015_828_MOESM1_ESM.docx]

***Supplement 1***

**Protocol for *HLA-Bw4* genotyping**

*HLA-Bw4* genotyping was performed using nested PCR-SSP. In the first step of analysis, a PCR reaction with specific primers for the whole *HLA-B* gene – F1: 5Bin157g 5’-ggg agg agc gag ggg acc gca g-3’; F2: 5Bin157c 5’-ggg agg agc gag ggg acc cca g-3’; R: 3BIn3-37 5’-gga ggc cat ccc cgg cga cct at-3’ – was done. PCR reaction was conducted in a 20 μl volume with: 2 μl PCR buffer, 40 mM MgCl, and 32 mM each dNTP, 1 pM of each primer, 2 U Taq Polymerase, and 200 ng of template DNA. Amplification was performed in the following conditions: 94° – 5 min, and 35 cycles (95° – 1 min; 64° – 1 min; 72° – 2 min); 72° – 7 min. 5 μl of PCR product was detected on 2% agarose gel with ethidium bromide. In the second step, amplification for *HLA-Bw4* and -*Bw6* alleles was done separately using the following primers for *Bw6*: R: 3BIn3-37 5’-gga ggc cat ccc cgg cga cct at-3’; F: Bw6F 5’-cct gcg gaa cct gcg cg-3’, for Bw4: R: 3BIn3-37 5’-gga ggc cat ccc cgg cga cct at-3’; Bw4F1: 5’-cct gcg cac cgc gct cc-3’; Bw4F2 5’-cct gcg gat cgc gct cc-3’; Bw4F3 5’-cct gcg gac cct gct cc-3’. PCR was conducted in a 10 μl reaction volume with: 2 μl PCR buffer, 25 mM MgCl, 20 mM each dNTP, 1.3 pM of each primer, 2 U Taq Polymerase, and 1 μl of 1000x diluted PCR product. Conditions were as follows: 95° – 5 min, 35 cycles (95° – 30 s; 52° (for Bw6) or 56° (for Bw4) – 55 s; 72° – 1 min and 72° – 7 min. The whole PCR product was identified by migration on 2.5% agarose gel stained with ethidium bromide.
