# Supplementary material for: KIR2DS5 in the presence of HLA-C C2 protects against endometriosis
Source: Immunogenetics. 2015 Mar 1;67(4):203–9. doi: 10.1007/s00251-015-0828-3 (PMC4357646; doi:10.1007/s00251-015-0828-3)
Supplement: Supplementary file 2 — (DOCX 13 kb) [file 251_2015_828_MOESM2_ESM.docx]

**Supplement 2.** Association of *KIR2DS5* with *HLA-C C2* in endometriosis

| **Group** | **HLA-C C2 +**  **N=230** | | **HLA-C C2 –**  **N=136** | |
| --- | --- | --- | --- | --- |
|  | 2DS5+ | 2DS5– | 2DS5+ | 2DS5– |
| Patients | 17 | 75 | 16 | 45 |
| Controls | 50 | 88 | 17 | 58 |
| OR | 0.4 | | 1.21 | |
| *p* | 0.005 | | 0.689 | |
| 95% CI | 0.21 - 0.75 | | 0.55 - 2.66 | |

Two-sided Fisher’s exact test was used to estimate differences between patients and controls.

N, Number of cases; *p*, probability; OR, odds ratio; 95% CI, confidence intervals
